# Persistent hiccup as one of the initial symptoms of leucine-rich glioma-inactivated-1 encephalitis: a case report

**DOI:** 10.1186/s12883-022-02797-w

**Published:** 2022-07-27

**Authors:** Lan Hou, Li Wan, Hongshan Li, Zhehui Wang, Hongzhi Guan, Haitao Ren, Pei Wang

**Affiliations:** 1 Department of Neurology, Baoding No.1 Central Hospital, 320 Northern Great Wall Street, Hebei Province 071000 Baoding, China; 2 Baoding City Key Laboratory of Neurological Diseases, Baoding, China; 3 College of Foreign Language, Hebei Agricultural University, Baoding, China; 4 Department of Neurology, Peking Union Medical College Hospital, Beijing, China

**Keywords:** Anti-leucine-rich glioma-inactivated 1, Hiccups, Case report, Encephalitis

## Abstract

**Background:**

Anti-leucine-rich glioma-inactivated 1 (LGI1) encephalitis, an autoimmune disorder, is characterized by faciobrachial dystonic seizures, epilepsy, memory deficits and altered mental status while hiccup is not commonly found in patients.

**Case presentation:**

A 62-year-old male was presented with slurred speech, abnormal gait, faciobrachial dystonic seizures and impaired cognition. Besides, the hiccup was one of the initial symptoms. His brain magnetic resonance images (MRI) revealed multiple lesions with left caudate nucleus, putamen, insula and left hippocampus involvement. Because a diagnosis of antibody-related limbic encephalitis was suspected, studies including an autoimmune profile were done by cell-based assays. After anti-LGI1 antibodies were detected in both cerebrospinal fluid and serology, pulse methylprednisolone and intravenous immunoglobulin were started and hence hiccups disappeared along with other symptoms.

**Conclusions:**

Clinicians should be aware that persistent hiccups might be one of the initial manifestations of LGI1 subtype of voltage-gated potassium channel complex antibody associated autoimmune encephalitis.

## Background

Anti-Leucine-Rich glioma-inactivated 1 (LGI1) encephalitis is an autoimmune disorder characterized by antibodies to the voltage-gated potassium channel complex (VGKC), known as limbic encephalitis, hyponatremia and faciobrachial dystonic seizures [1]. The co-existent hiccups have not been reported.

## Case presentation

**Fig. 1:**
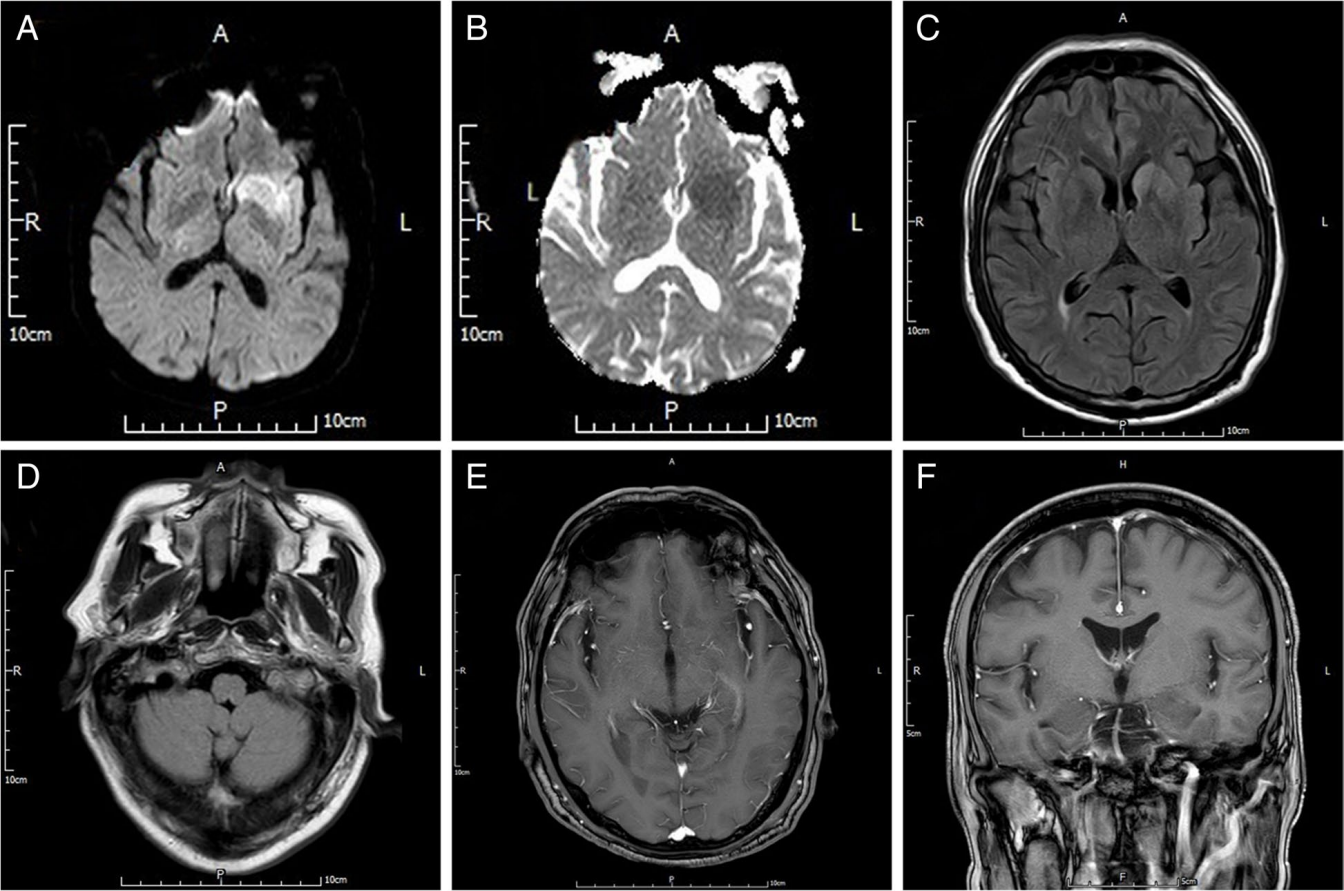
MRI sequences on the first admission. These images demonstrated irregular lesions in the left caudate nucleus, putamen and insula with restricted diffusion (**A**-**C**). Medulla oblongata was not affected (**D**). There was no obvious enhancement of the lesion on contrast-enhanced MRI (**E**, **F**). (**A**) DWI, **(B**) ADC, (**C** and **D**) FLAIR, and (**E** and **F**) T1 postcontrast

A 62-year-old male with a 2-year medical history of coronary atherosclerotic heart disease was admitted to our emergency department after acute onset of neurological symptoms. The patient presented with aggravated slurred in speech and abnormal gait in the prior 24 h, accompanied by nausea and vomiting. Additionally, he described having persistent hiccups of at least 2 h. Her cognitive decline was noted with a Montreal cognitive assessment (MoCA) score of 13 and a Mini-mental state examination (MMSE) score of 18 showing difficulties in areas of short-term memory, orientation and calculation. Emergency brain computed tomography (CT) revealed no acute process. The patient was transferred to department of neurology. The next day he developed symptoms of occasional jerky movements in the right face and twitching right leg, which would gradually increase in frequency. Continuous electroencephalogram monitoring was interpreted as background slowing without electrographic seizures. Brain magnetic resonance imaging (MRI) showed irregular lesions in the left caudate nucleus, putamen and insula with restricted diffusion (Fig. 1A-C). Medulla oblongata was not affected (Fig. 1D). No obvious enhancement was noted (Fig. 1E-F). Blood tests showed moderate hyponatraemia (118.04 mmol/L [reference range 137–147 mmol/L] ). The patient was therefore diagnosed with ischemic stroke and empirically treated with levetiracetam and sodium supplement. The patient was discharged after a nearly normal workup of serum sodium (135.03mmol/L) with recommendations to follow up with outpatient neurology. The patient was improved upon discharge.

**Fig. 2:**
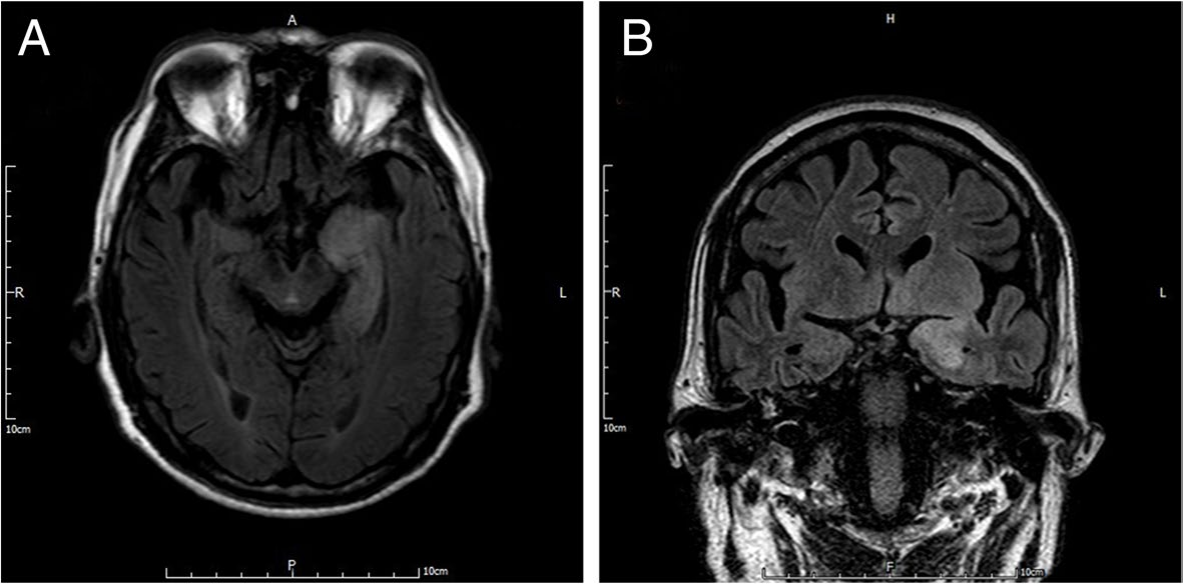
MRI sequences on the second admission. These images demonstrated new lesion which was hyperintensity on FLAIR sequences in the left hippocampus

Two days later, the patient was readmitted to the hospital for communication impairment, incessant hiccups and increasing frequency of jerking on both right face and upper extremity despite compliance with the prescription. His serum sodium was 120.22 mmol/L and MRI examination demonstrated new lesion in the left hippocampus (Fig. 2). Cerebrospinal fluid (CSF) workup results revealed a normal level in white blood cell count, glucose and protein. Anti-LGI1 antibodies were detected both in CSF and serology. CV2, anti-Ri, GAD65, Tr, Ma2, SOX1, Titin, Recoverin, amphiphysin, anti-Yo, Zic4, anti-Hu, and PKCγ antibodies in CSF and serum returned negative. CT scans of the chest, abdomen and pelvis demonstrated unremarkable for malignancy.

**Fig. 3:**
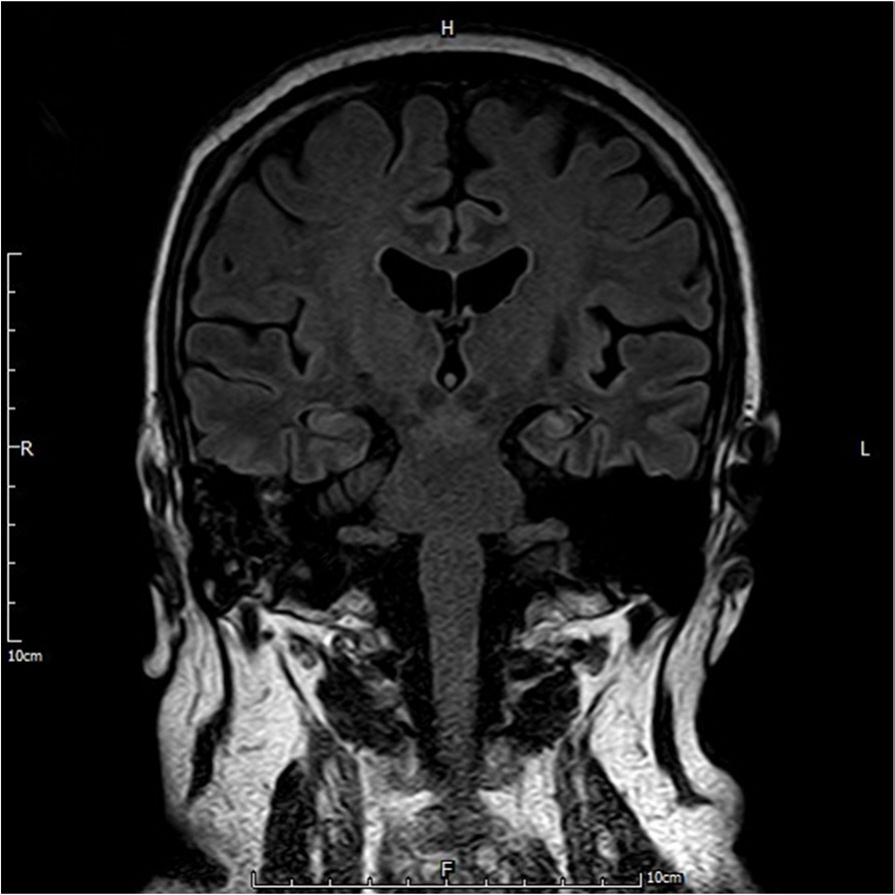
MRI sequence at 5-month follow up showed the left hippocampus was smaller than the contralateral side

His episodes were consistent with faciobrachial dystonic seizures. The patient was diagnosed anti-LGI1 antibody-mediated autoimmune encephalitis and treated with pulse methylprednisolone 1000 mg daily for 3 days, reduced by a half every 3 days. There were immediate improvements in his cognition and behavior and hiccups disappeared. However, neurological symptoms deteriorated when methylprednisolone was reduced to 120 mg. A course of intravenous immunoglobulin was therefore added. He was discharged several days later and continued on regular oral prednisolone. At 5-month follow up, he demonstrated obvious improvements in clinical symptoms. Besides, serum sodium was normal. But brain MRI showed the left hippocampus was smaller than the contralateral side (Fig. 3).

## Discussion and conclusions

We have presented an individual subsequently confirmed as LGI1-antibody encephalitis. Besides classic findings of LGI1 subtype of VGKC antibody-associated autoimmune encephalitis, this case illustrated a presentation of persistent hiccups which was gradually relieved after methylprednisolone and immunoglobulin treatment. The authors hypothesize that the hiccup might be one of the atypical symptoms of LGI1-antibody encephalitis.

Hiccups are considered as an episodic myoclonic activity of the diaphragm. It is generally believed to involve a reflex arc [2, 3]. Its afferents include phrenic, vagus, or T6-T12 sympathetic fibers. The efferent nerve is phrenic while the effector is the diaphragm. Although the hiccup center has not been fully clarified, it is postulated that the phrenic nucleus, respiratory center, brainstem reticular formation, and hypothalamus play an important role in the central nervous system. Factors that interact with this reflex arc have the potential to cause hiccups. The most likely affected in LGI1-antibody encephalitis is hypothalamus presented as hyponatremia. Although the main cause of hiccups in the central nervous system is thought to involve the brainstem especially the medulla oblongata and pons, supratentorial damages to the temporal lobe [4, 5], insular cortex [6, 7] and basal ganglia [8] caused by ischaemic stroke [6, 7], abscess [5, 8] or glioblastoma multiforme [4] have also been reported with the experience of intractable hiccups. For this patient, it was considered that the persistent hiccup was related to the lesions of the hypothalamus, basal ganglia, insula or temporal lobe. Besides, persistent hiccup is also one of the common manifestations of hyponatremia. There is a strong association between hyponatraemia and hiccups in hospitalized patients. It has been reported that chance of hiccups increases by 17 folds with a decrease by 10mmol/L of serum sodium [9].

In this patient, it is difficult to determine whether the hiccups arise from hyponatremia or temporal, basal ganglia, insula, hypothalamus involvement in this disease. However, hiccups appear with other symptoms and disappear after immunotherapy. It can be therefore concluded that persistent hiccups may behave as one of the atypical symptoms of LGI1-antibody encephalitis.

To summarize, among the clinical manifestations of LGI1-antibody encephalitis, persistent hiccups might be an early atypical symptom. Early immunotherapy can reduce complications and improve long-term outcomes.

## Data Availability

The datasets used and/or analysed during the current study are available from the corresponding author on reasonable request.

## Abbreviations

LGI1: Leucine-Rich glioma-inactivated 1
VGKC: Voltage-gated potassium channel complex
MRI: Magnetic resonance imaging
CT: Computed tomography
CSF: Cerebrospinal fluid
